# Personalized medicine: evidence of normativity in its quantitative definition of health

**DOI:** 10.1007/s11017-016-9379-3

**Published:** 2016-09-16

**Authors:** Henrik Vogt, Bjørn Hofmann, Linn Getz

**Affiliations:** 1 General Practice Research Unit, Department of Public Health and General Practice, Norwegian University of Science and Technology, Trondheim, Norway; 2 Section for Health, Technology, and Society, Norwegian University of Science and Technology, Gjøvik, Norway; 3 Centre for Medical Ethics, University of Oslo, Oslo, Norway

**Keywords:** Health concepts, Medicalization, Naturalism, Normativism, Systems medicine, Personalized medicine

## Abstract

Systems medicine, which is based on computational modelling of biological systems, is emerging as an increasingly prominent part of the personalized medicine movement. It is often promoted as ‘P4 medicine’ (predictive, preventive, personalized, and participatory). In this article, we test promises made by some of its proponents that systems medicine will be able to develop a scientific, quantitative metric for wellness that will eliminate the purported vagueness, ambiguity, and incompleteness—that is, normativity—of previous health definitions. We do so by examining the most concrete and relevant evidence for such a metric available: a patent that describes a systems medicine method for assessing health and disease. We find that although systems medicine is promoted as heralding an era of transformative scientific objectivity, its definition of health seems at present still normatively based. As such, we argue that it will be open to influence from various stakeholders and that its purported objectivity may conceal important scientific, philosophical, and political issues. We also argue that this is an example of a general trend within biomedicine to create overly hopeful visions and expectations for the future.

## Introduction

The emerging concept of systems medicine has become an increasingly prominent part of the movement towards personalized (or precision) medicine in the wake of the sequencing of the human genome 15 years ago [1]. As a clinical framework, it is often promoted as ‘P4 medicine’ (predictive, preventive, personalized, and participatory) [2]. Systems medicine is the medical application of systems biology [3]. The defining feature of systems biology is its ambition to use computational *in silico* modelling of genomic and other big data from all levels of biological organization in order to gain an integrative or ‘holistic’ understanding of living organisms *as whole systems* [4, 5]. For this purpose, its modelling strategies are adopted from mathematics, engineering, physics, and computer science [6].

Proponents of systems medicine have in the past five years made increasingly bold and specific promises that systems medicine will be able not only to demystify disease by elucidating underlying causes but also to quantify wellness (health) and develop—for the first time in history—a quantifiable metric for wellness [7, 8]. This promise is also related to the ambition to develop a personalized medicine that can account for variation among individuals: the metrics are promised to ‘let us assess wellness and its dynamics for each individual’ [2, p. 7]. The primary source of these promises is biologist Leroy Hood and his collaborators at the Institute for Systems Biology (ISB); P4 Medicine Institute (P4MI) in Seattle, USA; and affiliated research institutions in Europe.

Claims about a scientific metric, which would effectively function as a scientific benchmark for health, are clearly relevant for fundamental debates in the philosophy of medicine between two main positions, naturalism and normativism:

According to naturalists, disease and health are descriptive concepts that can be used to define the objective and real state or condition of a person. These concepts are strictly neutral to any personal or social values. According to the normativists, however, these concepts depend upon personal and social values. [9, p. 63]

The claims made about demystifying disease and quantifying wellness (or health) appear wholeheartedly naturalistic, in particular because the promise is that the new metric eliminates the purported *vagueness, ambiguity,* or *incompleteness* of previous definitions of health [8, 10]. We interpret this claim as implying a definition that is not influenced by personal or social values. This would fulfil a grand ambition of medicine, that is, to free itself not only from the fallible perceptions of patients but also from the biased interpretations of physicians [11]. The claim of a non-vague, non-ambiguous, and complete definition of health is a strong claim because many philosophers, scientists, and clinicians hold disease concepts, and especially concepts of health, to be essentially normative [12].

This article is grounded in extensive research on systems (P4) medicine as a framework for primary care medicine [13–15], including in articles obtained from PubMed searches on ‘systems medicine’, ‘P4 medicine’, and the author ‘Hood L’, as well as various Google searches and lists of publications, patents, and news at the ISB and P4MI websites [16–18]. A main premise for our argument is that—as far as we can see—Hood and coworkers have hitherto provided little explicit empirical evidence or conceptual work to support their promise of a metric for wellness. In this article, we therefore assess whether the promise of a metric for wellness—one that can for the first time eliminate the ‘vagueness’, ‘ambiguity’, or ‘incompleteness’ of currently existing health concepts—is reasonable. We do this by asking to what extent P4 systems medicine advocates have so far been able to provide a *non-normative* way of defining health.

By examining in particular a US patent assigned to the ISB that describes a method for assessing health and disease [19], and referring also to concrete examples of ISBs work with diagnoses, we argue that systems medicine has so far not provided a *non-normative* way of defining health. On the contrary, its definition of health and disease would be open to influence by various stakeholders and their goals and values, while the promise of scientific objectivity may conceal this normativity and thus obscure important issues. One potentially problematic issue is that influence by stakeholders on the definition of health and disease may lead to an overmedicalization of problems that should not be understood or tackled in medical terms, or at least not in reductive biomedical terms. Medicalization is then understood as the process by which aspects of human life are defined in medical terms and underlain medical control [20]. Additionally, we argue that our findings can be viewed in light of a tendency within biomedicine to hype and oversell its capacities in order to create visions and expectations of what it will be able to achieve [21]. Although the creation of such visions and expectations of the future is not part of the core, scientific work, it is nonetheless an important part of biomedical culture, influencing actions and choices in science, research funding, healthcare, and society at large. As such, they should be examined critically. In this regard, it must be noted that those who make these promises are powerful. Leroy Hood is widely considered a leading visionary in biotechnology [22]. According to its website, the ISB is ‘ranked 4th in the world for research impact’ in its field [23]. Its Hundred Person Wellness Project, which pioneered clinical research on P4 medicine, was prominently featured in *Nature* [24].

## Promises of a metric for wellness

In order to contextualize our argument, we begin by briefly outlining the promises that are being made about a metric for wellness. This illustrates how an impression is created that a transformative objective naturalism is in the scientific pipeline.

In a striking development, we are currently witnessing promises of turning the concept of ‘wellness’ into a *comprehensive* (as in dealing with *all aspects* of a phenomenon) yet *scientific* definition of health. As an example, Alfredo Cecario et al. recently stated that ‘wellness, as a status to be achieved and maintained in our lives, getting longer and hopefully healthier, is the new and comprehensive declination of “health” itself, leading the shaping of research and research policy in the health domain worldwide’ [25]. These claims are being made in the context of biomedical entrepreneurialism, consumerism, and an increasing focus on disease prevention through proactive enhancement of wellness in healthy people, as opposed to reactive cure of disease [21, 26, 27]. The ISB’s annual report for 2014 states:

P4 medicine has two central thrusts—improving wellness and helping to avoid disease. Perhaps 97% of society’s healthcare resources are spent on disease and very few on wellness. Accordingly, wellness—and how to enhance it and extend it—has not been studied very thoroughly by scientists. ISB proposes to change this by taking a systems-approach to understanding wellness—and thereby make it scientific. [28]

It is this systems-approach that is methodologically promised to allow demystification of disease and quantification of wellness:

Through systems biology and systems medicine, new computational models of multilevel biological networks are being established. These models decipher biological complexity by showing how all elements in biological systems interact with each other to produce health and disease states. They are being systematically tested and adjusted to become increasingly powerful predictors of each individual’s personal experience of health and disease. These models not only demystify disease, they also quantify what it means to be healthy. [7]

As an extension of these promises of quantifying ‘what it means to be healthy’ and predicting future experience of health, systems medicine is also promised to be able to provide *a quantifiable metric for wellness*.

In 2014, the ISB launched the ‘Hundred Person Wellness Project,’ a pilot phase of a planned 100 K Wellness Project, which is proposed to longitudinally study 100,000 people to demonstrate the clinical utility of P4 medicine [24]. With regard to our argument, a prime objective for this project is ‘to mine the data from those individuals who maintain wellness (or exhibit increased wellness) for metrics that will, for the first time, provide a quantitative foundation for the currently vague and incomplete definitions of wellness’ [8]. Notice the reference to individuals who ‘exhibit increased wellness.’ By a metric for wellness, Hood and coworkers seem not only to indicate a metric that defines normality, but health as something positive, something more than mere absence of disease. Mauricio Flores et al. refer to this positively defined wellness as a ‘wellness space’ in which new companies can operate to optimize the wellness of consumers [7]. Hood and coworkers sum up the promise of a quantitative definition of health as follows:

While wellness and prevention may have great conceptual appeal, there are relatively few widely accepted, quantifiable metrics to define ‘wellness’. … Thus, there is a real need to define and systemize quantifiable wellness metrics, with longitudinal data that supports their validity and clinical usefulness. Moreover, we believe that we can eventually generate a multiparameter metric for wellness—by employing data from individuals exhibiting wellness over an extended period of time. It will reflect both the psychological and physiological aspects of wellness, thus quantifying wellness—a concept to date that has been defined in vague and ambiguous terms’. [10]

In other words, the ambition is effectively to quantify, control, and thus medicalize health itself [13].

## The patent: a multiparameter analysis for predictive medicine

In order to answer our question of whether the promise of a non-vague, non-ambiguous metric of wellness holds, we will here examine a US patent for a ‘multiparameter analysis for predictive medicine’ listed on the ISB website [19]. A patent is a particularly valuable research material in the context of personalized medicine because it directs scientists to be concrete as to what they will actually do to realize their promises, which may be unclear in publications stating their bold visions. The patent in question refers to the concept of a ‘health-associated reference expression region’, which reflects health, and how such a region can be determined. We find this to be the most concrete and relevant description to date of how health or wellness will actually be defined in P4 systems medicine, and to be the closest thing yet to a metric for wellness found in the literature. We will analyze the patent stepwise and in some detail.

### Rationale and purpose

The patent describes a method for diagnosing the health state (understood as a state of disease or a state of health) in an individual, based on measurements of a sample of molecules that reflect several levels of gene expression. Gene expression is the process by which sequences of DNA are transcribed into RNA and subsequently translated into protein molecules that enable function (or dysfunction) in organisms. The patent explains the rationale of the invention as follows:

gene expression patterns are expected to change when an individual has a disease. Information on gene expression patterns thus provides a basis for efficient and accurate diagnostic methods based on changes in gene expression in various diseases. The exploitation of genomics and proteomics information thus requires methods that can account for the large number of genes and complexity of gene expression patterns useful for medical applications. Fully exploiting genomics and proteomics information for medical applications requires methods that can accurately and efficiently monitor complex changes in gene expression patterns both at the mRNA and protein levels. Thus, there exists a need for methods to efficiently diagnose a disease based on gene expression patterns in an individual. The present invention satisfies this need and provides related advantages as well. [19, p. 10]

The patent states that the molecular measurements needed in the method can be conveniently performed by sampling a specimen from an individual, such as readily accessible white blood cells that ‘can provide a window into many physiological systems’ [19, p. 11]. The patent also states that the method may eventually involve measurements of thousands of molecules [19, p. 19]. These statements are congruent with claims made by Hood and coworkers in other publications that they will make blood ‘a window for monitoring health (wellness) and disease’ [29] and eventually construct ‘a small handheld device that can prick your thumb, measure 2,500 organ-specific proteins, send this information to a server for analysis and feedback the information on the state of your 50 organ systems’ [2]. The patent describes that the method of the invention will involve computational analysis that ‘can include linear, non-linear, and/or multivariate calculations from fields including mathematics, statistics, and/or computer science’ [19, p. 18]. In other words, this is clearly a patent for a method that is in line with the general aim of systems biology: to tackle the challenge of biocomplexity that has become increasingly apparent in biomedicine [30]. Indeed, we read the patent as a step for the ISB towards securing rights to P4 systems medicine’s method of diagnostics and prognostics itself.

### Assessment of health versus disease

How does the patent further explain how health (wellness) and disease can be assessed? The method proposed involves ‘comparing the expression levels of a sample of molecules … from an individual with one or more health-associated reference expression regions of the sample of molecules’ [19, p. 10]. The central concept here is *health-associated reference expression region*, which defines health. If an individual is found to have expression levels within such a health-associated reference expression region, then this is taken to indicate that the person is in a state of health (‘a reference health state’), whereas levels outside is indicative of ‘a potential disease state in the individual or of a predisposition to developing a disease’ [19, pp. 1, 11, 12, 39]. According to the patent, ‘the determination of a health-associated reference expression region … provides a central repository of information’ [19, p. 12]. Although the term ‘metric’ is not used explicitly in the patent, we interpret the status in developing such a repository as representative of the status of developing a scientific definition of health in systems medicine, at least in terms of what has been published.

**Fig. 1:**
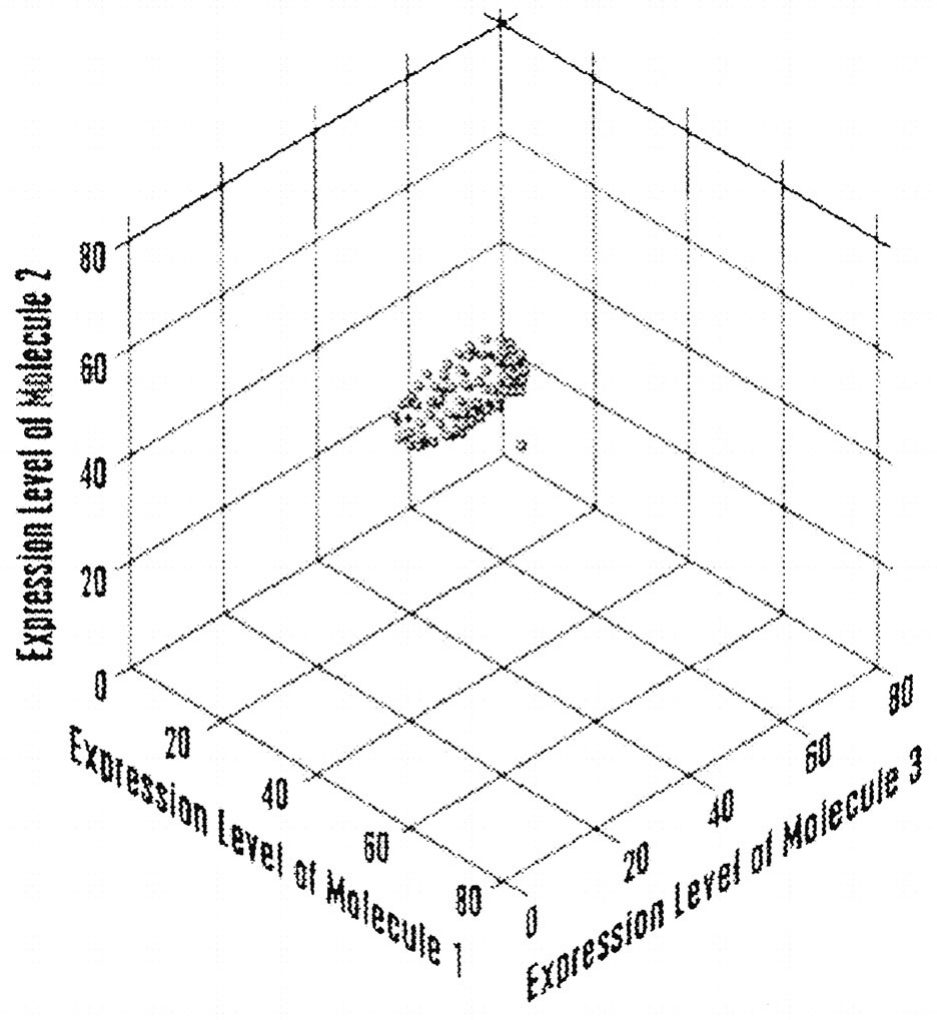
Facsimile of figure taken from the patent ‘Multiparameter Analysis for Predictive Medicine’ assigned to the Institute for Systems Biology [19, p. 4]. The patent text explains that the figure shows ‘a schematic diagram of a hypothetical health-associated reference expression region in three-dimensional space. In this case, each coordinate point represents the expression levels of three molecules in an individual, which define a three-dimensional coordinate point. A three-dimensional ellipsoid represents a health-associated reference expression region in three-dimensional shape space. Also shown is an individual having coordinate points that lie outside the health-associated reference expression region…. A similar analysis can be applied in *n*-dimensional space, where *n* is the number of molecules in a sample of molecules…. In such a case, a health-associated reference expression region is defined in *n*-dimensional shape space based on the *n*-dimensional coordinate points of a reference population of individuals’ [19, p. 23]

In the patent, the method is more specifically described as involving the measurement of multiple parameters in a specimen (e.g., blood) from an individual [19, pp. 10, 11]. The number of parameters is called ‘*n*’. A multidimensional analysis of health or disease is then conducted wherein the combination of expression levels of *n* molecules from an individual can be represented as a coordinate point in a space of *n* dimensions [19, pp. 14, 17]. The ‘health-associated reference expression region’ is a multidimensional region in ‘*n*-dimensional shape space’ against which the coordinate point of the individual can be compared [19, pp. 14, 23]. This shape-space may be graphically represented, as illustrated in Fig. 1. The explicit reference to health as a region in a ‘space’ is reminiscent of Hood and coworkers’ reference elsewhere to a ‘wellness space’ in which health can be optimized by novel, lucrative companies [7].

### Establishment of the definition of health

A crucial question now arises: How is the health-associated reference expression region concretely defined and established?

According to the patent, the health-associated reference expression region is to be statistically determined from the molecular expression levels in a population of *reference individuals* [19, pp. 11, 12, 14]. In other words, health is defined by studying populations of individuals whose functioning is taken to be representative of health.

But how are the individuals that comprise this reference population to be identified? The patent states that they will be selected according to certain *reference criteria of healthy individuals*. How then will these criteria be established? The patent states that, ‘*One skilled in the art* can readily determine desired criteria for the reference population and *select individuals fitting the desired criteria*’ [19, p. 14] (emphasis added). Furthermore, it states that, ‘*One skilled in the art* can readily determine if an individual is in good health based on *subjective feelings of wellbeing* of the individual and objective signs of disease in an individual’ [19, p. 14] (emphasis added). With regard to deviations from health, the patent also states that, ‘*One skilled in the art* can readily determine if an individual has signs or symptoms associated with a particular disease. Moreover, *one skilled in the art* can also readily determine whether an individual has signs or symptoms that are recognizable as lying outside the condition of a healthy individual’ [19, p. 16] (emphasis added).

## Discussion

### Normative definitions of health and disease

The following, then, is how the health-associated reference expression region is defined. Firstly, ‘*one skilled in the art*’, that is, someone, an agent (or several agents), selects a population that is taken to represent health according to desired criteria for health. Secondly, this agent selects according to signs and symptoms that may also include *subjective feelings of wellbeing*. This clearly shows that the definition of health used in the patent will not be free of social or personal values.

If our premise that this health-associated reference expression region is the closest thing to a ‘metric for wellness’ in systems medicine holds, our finding weakens the claim that systems medicine is about to provide a non-normative metric of wellness that will eliminate the vagueness, ambiguity, or scientific incompleteness of previous definitions. Despite promises of a transformative scientific objectivity, we find at its foundation *the art of medicine* [31]. Importantly, this reliance on professional and cultural judgement in defining who is healthy and who is unhealthy is not emphasized in other publications of Hood and coworkers. Instead, we find an emphasis on the prospect of a new, scientific definition of health [2, 7, 8, 10]. In this way, a naturalistic, bio-molecular makeup may cover its normative foundation and hence potentially mislead political and professional aspirations to maximize personal and population health in a sustainable and responsible manner [32].

It could of course be argued that the method described in the patent is in keeping with a form of naturalism, as naturalists do not necessarily deny that a person or society may evaluate a condition as good or bad in addition to an objective description. Their point is rather that ‘the basic scientific description and the evaluation are … two independent matters’ [12]. Normativists, on the other hand, do not necessarily deny that science can provide crucial knowledge that can be used to define disease and health, but hold that these judgements are, in the end, normative. We do not aspire to provide a definitive answer to these vast and complex issues [33]. For the purposes of our argument, it is enough to state that the patent does not point towards a metric of health that eliminates the vagueness and ambiguity in the process of defining what is wellness and what is disease. On the contrary, it brings matters back to the fundamental question of how one presents a reference for health.

‘One skilled in the art’—or ‘person skilled in the art’—is admittedly also a legal term in patent law used to denote a person with a certain competence in a field [34]. We still interpret the patent, however, as indicating real human agents. Theoretically, the term ‘person skilled in the art’ might not translate into human agents with values and conflicts of interest. But if that is so, the patent does not specify how a reference population displaying health can be objectively chosen. Regardless of whether ‘a person skilled in the art’ refers to a human expert, an algorithm, or a combination of both, selection of the reference population clearly presupposes a series of value judgements.

### A metric open to pressure

An important implication of the metric of wellness being normatively determined is that this would open it up to influence by various agents and their goals and values. This is not very different from ongoing debates on health and disease. For example, when various organizations fight for their condition to be accepted as a ‘real’ biomedical disease (i.e., caused by chemicals, infectious agents, immunological reactions, or other ‘physical’ processes at the molecular or cellular levels), they appeal to their own goals and values [35–37]. An interesting feature of the systems medicine case is that Hood and coworkers so clearly define their approach as naturalistic even though it obviously still is normativistic. In practice, any agent who influences systems medicine and underlying cultural ideas of what constitutes health and disease (e.g., industrial economic interests and patient consumer demand) might have an impact on the reference health state. This is not to say that systems medicine cannot develop useful, quantitative models that function as correlates of health and disease or be informative in the definitional process. It just means that there is no evidence to prove that this process will now be purged of normative and subjective considerations. Such considerations may in some instances be quite uncontroversial. The definitional process, however, may also involve ethical problems and professional debate. As one important example, the definitional process may be taken to generate overmedicalization with unacceptable potentials for waste and harm [20].

### Examples from ISB research

To further illustrate potential problems with providing quantitative correlates to constructs of health and disease that have, in the first place, already been defined by normative agents, we will now consider three diagnoses that Leroy Hood’s ISB has worked on or will work on. All of these diagnoses involve controversies and pressure from various agents as to how they should be defined.

The ISB has been working on a blood test to diagnose post-traumatic stress disorder (PTSD) [38]. Citing discovery phase research, Hood has claimed that ‘this is the first time ever that we’ve converted a disease that heretofore had been diagnosed psychologically into a quantitative blood test. And I think we will be able to do this for all neuropsychiatric diseases and fundamentally change the way we approach those diseases in the future’ [39] (see 45 minutes into the cited video).

If a disease is ‘converted’ from being diagnosed ‘psychologically’ to being diagnosed quantitatively, then it may seem as if the diagnosis almost ‘magically’ becomes objective and non-normative. However, as an editorial in *The British Journal of Psychiatry* explains, no distinct cause has been found for this syndrome. Its only distinctiveness lies in the very set of (psychological) symptoms and signs that has already been defined as abnormal by groups of psychiatrists [40]. The validity and ethical implications of this diagnostic entity may therefore be questioned like many other medical and psychiatric categories. As the editorial also points out, there have been different opinions as to where the line between normal and abnormal should be drawn in PTSD, and the category has been the subject of ‘diagnosis creep’ where it has ‘been extended to an increasing array of events and human reactions across diverse cultures’ [40]. Providing a molecular correlate to such a fluid entity—however sensitive and specific it may be in ‘confirming’ that the patient ‘has’ the disease as presently defined—can hardly be seen as ‘demystifying’ the disease, that is, eliminating its vagueness or ambiguity and instead providing a definitive scientific description.

The ISB has also worked on the genetics of the bipolar disorder syndrome [41]. This category is also controversial and under criticism for being influenced by vested interests and for overmedicalizing mood fluctuations in both adults and children. It too is defined, in the first place, by groups of psychiatrists with self-interests and bonds to the pharmaceutical industry [42, 43].

The most concrete and serious example of challenges involved in generating quantitative correlates to categories of health and disease pertains to so-called ‘chronic Lyme disease’. In 2015 the ISB announced the ‘3-year Wilke Lyme Disease Project’ to be led by Leroy Hood, with 2.13 million USD in funding from The Wilke Foundation and The Bay Area Lyme Foundation. According to Hood and the ISB, systems biology approaches will ‘develop diagnostics and a deeper understanding of chronic Lyme’, being also ‘the only way through the massive complexity surrounding Lyme disease’ [44]. Lyme disease is an infectious disease caused by the bacterium *Borrelia burgdorferi*, which is transmitted by a tick vector. This is uncontroversial within infectious medicine. However, ISB’s focus will evidently be on so-called ‘chronic Lyme’, a proposed chronic version that is purportedly also caused by tick-borne microbes but a ‘highly complex and often misdiagnosed disease that can be debilitating to those who do not respond to a standard course of antibiotics’ [44].

In this case it seems that the ISB is rather uncritically adopting the position of a group of physicians calling themselves ‘Lyme literates’ and Lyme advocacy groups who claim that ‘chronic Lyme’ exists as a ubiquitous disease that is very hard to diagnose and cure, a disease that should be treated with long-term antibiotics and that purportedly explains a range of highly prevalent non-specific symptoms (e.g., fatigue and pain). However, the existence of ‘chronic Lyme’ understood in this way is highly controversial. Experts in infectious medicine have tried to underscore that it is an unfounded construct to explain various so-called ‘medically unexplained symptoms’, which have often been thought to have complex causes where personal or social-level factors play an important role [36, 45, 46]. They have warned that the Lyme advocacy movement, which also sponsors research, has ‘created a pseudoscientific and alternative selection of practitioners, research, and publications and have coordinated public protests, accused opponents of both corruption and conspiracy, and spurred legislative efforts to subvert evidence-based medicine and peer-reviewed science. The relations and actions of some activists, medical practitioners, and commercial bodies involved in Lyme disease advocacy pose a threat to public health’ [36].

This is the terrain into which the ISB ventures with its quantitative method. Hood and the ISB seem to think that measuring and analyzing molecular dynamics in people who have been labelled with ‘chronic Lyme’ will inevitably provide a ‘deeper’ understanding of the phenomenon. But this is far from obvious. When the ISB uncovers molecular correlates to the spectrum of suffering experienced by this group (which they eventually will, one assumes, given that every human state or condition has molecular aspects), it might contribute new descriptive knowledge but not necessarily better causal understanding. If ISB researchers end up identifying molecular correlates to a non-valid construct, and these correlates are mistakenly taken as proof that ‘chronic Lyme’ exists as a chronic, non-psychological, microbially caused disease, then this would be counterproductive. It might divert attention away from other research, distract from other causal factors at other levels, and lock patients in a false assumption that their persistent problems are microbially or biomolecularly caused.

For the purposes of our argument, these three examples of PTSD, bipolar disorder, and chronic Lyme disease illustrate that—as in the patent—the ISB seems in practice to accept and develop quantitative correlates to constructs of health and disease that are already defined by other agents and continually open to normative pressure from various stakeholders (e.g., pharmaceutical industry or patient advocacy groups funding research). It is a conceptual fallacy to assume that a purely quantitative systems medicine alone eliminates the ‘vagueness’ or ‘ambiguity’ of these concepts or renders them perfectly objective. Instead, it might, under the cover of objectivity, camouflage some very profound scientific and philosophical questions concerning the nature and origins of wellness, illness, and disease. It may also stifle important debates about what physicians and scientists are actually doing when, for example, they label someone as ‘at risk’ or as afflicted by ‘bipolar’, ‘PTSD’ or ‘chronic Lyme’. Moreover, by selecting a reference of health and making any deviation of functioning from that reference the responsibility of the individual or a public health system, one effectively labels an unknown segment of the population as deviants with respect to optimal health, thereby potentially also inflicting iatrogenic harm such as anxiety or altered self-image [47]. It is far from obvious that this important definitional work can be left to scientists at the ISB or other abstract agents ‘skilled in the art’.

### Biomedicine: a culture of dreams

Our analysis illustrates another important aspect of modern biomedical practice. Sociologist Richard Tutton has highlighted how stakeholders in biomedicine in general, and personalized medicine in particular, regularly produce visions of what they will achieve in the future in order to create an attractive anticipation of what is to come [21]. This can be seen as an essential part of the biomedical culture: providing goals to pursue. Such visions may, or may not, be well grounded in scientific evidence or philosophy. However, a tendency towards hyping seems to have gained broad cultural legitimacy as part of a general belief in progress. Crucially, such hype may inform choices, even in disciplines which otherwise have high standards for scientific rigor. The promises of personalized medicine may serve important non-scientific goals (e.g., to attract funding, political support, and popular interest) and should therefore be questioned as part of the decision-making process. As sociologist Catherine Waldby has pointed out, such biomedicine may create theoretical images of human bodies that ‘comply with medicine’s fantasies of perfect management’ [48]. A metric is a culturally intelligible and scientifically significant metaphor. The thought of developing a metric that renders the functioning of each person eminently quantifiable can be interpreted as contributing to such an attractive vision of ‘perfect management’. However, creating false hopes and expectations is also ethically problematic because it may misinform actions of patients, politicians, and other agents [49].

### Personalized versus scientific medicine

The vision and promise of personalized medicine is that it will account for variation among individuals and thus relieve medicine of the problems associated with a ‘one size fits all’ practice [21]. The newly started company Arivale, which springs out of the ISB, even promises its clients ‘extreme personalization’ [27]. As a final point in our discussion, we find the patent relevant for considering this core promise. As mentioned in the introduction, the metrics of wellness are also promised to let us ‘assess wellness and its dynamics for each individual’ [2].

With regard to such personalization, the patent does mention how the reference population may be stratified into sub-populations according to genetic and other criteria such as diet, drug intake, age, gender, and physiological states (e.g., exercise, rest, or sleep) [19, p. 14]. The individual may then be compared to a sub-population of reference individuals that show similar characteristics. What the patent first and foremost documents, however, is how so-called ‘personalized medicine’ still relies on population-based methods. The provision of care according to population-based, scientific metrics for wellness is in tension with the aim and promise of accounting for variation among individuals.

In order to truly grasp the immensity of the challenge of accounting for variation among individuals, one must consider that molecular or physiological variation, which is the main focus in P4 systems medicine, is not independent of variation at the level called ‘psychological’ or ‘cultural’. Physiological or molecular variation should not be conceptualized as non-psychological, non-normative, or not influenced by personal experience and values [14, 50, 51]. Given the general, physicalistic premise that mental or social events are always necessitated by molecular interactions, molecular variation will, in the end, turn out to be no less complex than variation among human beings in their cultural and environmental context. As empirical research has also shown, the molecular and physiological levels are influenced by personal and inter-relational experience, which are in turn constrained by values and norms [52–56].

It remains unclear how P4 medicine can provide ‘extreme personalization’—as in account for variation resulting from individual experience at the personal and social levels—and, at the same time, employ its population-based metric.

## Conclusion

Proponents of P4 systems medicine have made bold claims and promises about a coming metric for wellness that will eliminate the vagueness, ambiguity, or incompleteness of earlier definitions. By analyzing a patent relevant for evaluating these promises and by studying the progress in developing such a metric, we have identified clear normative elements. These are concealed under the apparently value-neutral professional judgments of agents ‘skilled in the art’ of selecting a healthy reference population. If the method described in the patent is representative of a future metric of health, then this metric would be vulnerable to influence by various stakeholders. As we have also argued through examples taken from the research of the ISB, it is a fallacy to assume that providing a quantitative correlate to a construct that is already normatively defined automatically makes it objective, purely scientific, or non-normative. This purported scientific objectivity might obscure influence from stakeholders and important scientific, ethical, and political challenges to defining health and disease, such as concerns of overmedicalization. The promise of a metric for wellness may be seen as an example of a cultural trend in biomedicine for creating overly hopeful expectations for the future. We have pointed out deep tensions between the suggested scientific and objective metric of health and the promise of a medicine that is truly *personalized*. To pursue a scientific, non-ambiguous, non-vague definition of human wellness, which will allow one to assess health in each person—and to seek to operationalize and then patent methods for assessing this concept—seems a contemporary example of what biologist René Dubos in 1959 called ‘the mirage of health’. It is a view of biomedicine as perpetually chasing the utopian dream of pinning-down the constantly moving target of health, a phenomenon that ultimately answers to all the dynamic complexities of human life, mind, and culture [57].
